# Metabolic and Proteomic Profiles Associated with Immune Responses Induced by Different Inactivated SARS-CoV-2 Vaccine Candidates

**DOI:** 10.3390/ijms231810644

**Published:** 2022-09-13

**Authors:** Shouzhi Yu, Yao He, Wenheng Ji, Rong Yang, Yuxiu Zhao, Yan Li, Yingwei Liu, Ling Ding, Meng Ma, Hui Wang, Xiaoming Yang

**Affiliations:** 1 Beijing Institute of Biological Products Company Limited, Beijing 100176, China; 2 China National Biotec Group Company Limited, Beijing 100024, China

**Keywords:** COVID-19, metabolism, proteomics, vaccines

## Abstract

Since the emergence of severe acute respiratory syndrome coronavirus 2 (SARS-CoV-2) in late 2019, the virus has been mutating continuously, resulting in the continuous emergence of variants and creating challenges for epidemic prevention and control. Here, we immunized mice with different vaccine candidates, revealing the immune, protein, and metabolomic changes that take place in vaccines composed of different variants. We found that the prototype strain and Delta- and Omicron-variant inactivated vaccine candidates could all induce a high level of neutralizing antibodies and cellular immunity responses in mice. Next, we found that the metabolic and protein profiles were changed, showing a positive association with immune responses, and the level of the change was distinct in different inactivated vaccines, indicating that amino acid variations could affect metabolomics and proteomics. Our findings reveal differences between vaccines at the metabolomic and proteomic levels. These insights provide a novel direction for the immune evaluation of vaccines and could be used to guide novel strategies for vaccine design.

## 1. Introduction

The emergence of coronavirus disease 2019 (COVID-19), caused by SARS-CoV-2, has had a disastrous impact on people all over the world, causing more than 6 million deaths [1]. Vaccines are critical cost-effective tools for preventing COVID-19 by protecting people from infection [2,3]. At the time of writing, 12 billion doses of the COVID-19 vaccine have been administered globally, with 66.5% of the population having received at least one dose. Vaccination has significantly reduced hospitalizations, severe cases, and deaths, and helped to protect the global public health system [4]. Studies have confirmed that inactivated vaccines can induce high levels of neutralizing antibody titers and are effective against COVID-19 [5].

Over the past two years, multiple SARS-CoV-2 variants have emerged globally, including Alpha (B.1.1.7), Beta (B.1.351), Gamma (P.1), Delta (B.1.617.2), and Omicron (B.1.1.529) [1,3,6]. These variants show immune evasion characteristics and increased transmission capacities, leading to the possible ineffectiveness of vaccines [7]. The evolutionary trajectory of SARS-CoV-2 remains uncertain, so the genetic and antigenic properties of future variants cannot be predicted. The emergence of new variants may overturn the great efforts made so far to limit the spread of COVID-19 [1]. In order to maintain the broader immunity offered by the vaccine, it is necessary to continuously update its components and discover new immune markers or antigens. Systems vaccinology is used to characterize vaccine-induced immune responses and provide insights into the rational design of new vaccines through multiple omics technologies [8,9]. Vaccination stimulates the innate and adaptive immune cell responses of the body and causes changes in proteomics and metabolic pathways [10]. As an important part of systematic vaccinology, proteomics and metabolic research have opened up a new way in which to understand the molecular mechanism of vaccine-induced immunity [10,11]. However, there is a lack of omics-techniques-related studies on the body after immunization with vaccine candidates for different COVID-19 variants.

Here, we analyzed the neutralizing antibody levels, serum proteomics, and metabolomics of mice vaccinated with different COVID-19 inactivated vaccine candidates. We demonstrated that immunization with different COVID-19 inactivated vaccine candidates induced cellular and humoral immune responses in mice, including antibody production and cytokine production. In addition, our results reveal the metabolic and protein profiles induced by different vaccine candidates.

## 2. Results

### 2.1. Immunogenicity Analysis

**Figure 1:**
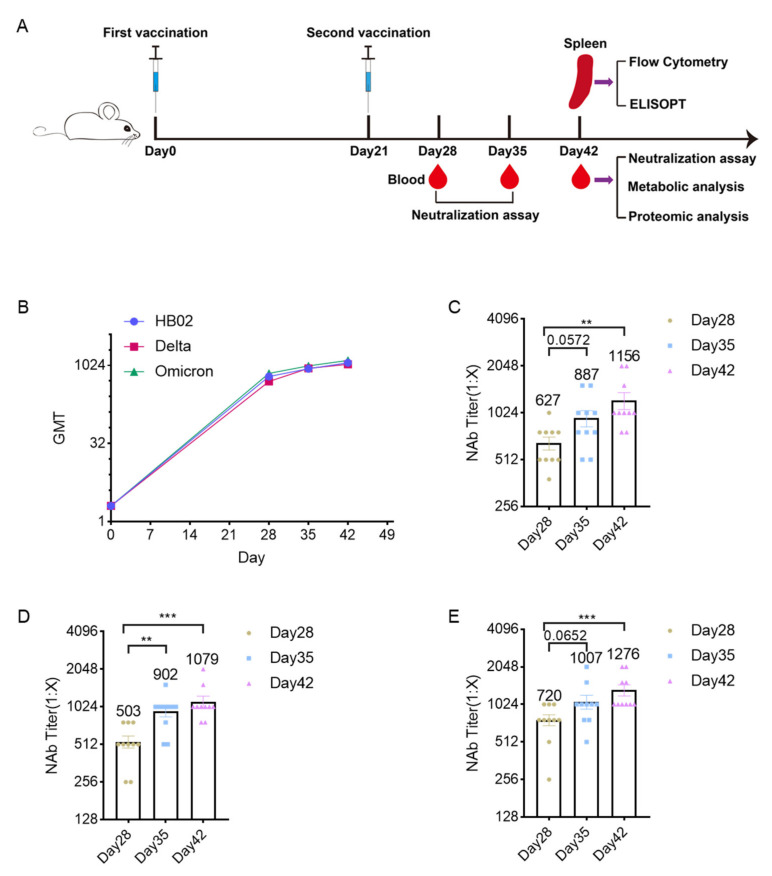
Analysis of neutralizing antibody levels of different inactivated vaccines. (**A**) Experiment schematic—the BALB/c mice were injected with different vaccines on day 0 and day 21, and the blood was collected on day 28/35/42 after the first immunization. The serum from day 28/35/42 was used for the neutralization assay; additionally, the serum from day 42 was analyzed via metabolic and proteomic analysis. Additionally, the spleens collected on day 42 after immunization were used in flow cytometry and ELISPOT. (**B**) The GMT levels of the HB02, Delta, and Omicron vaccines against their respective strains on day 28/35/42 after immunization; (**C**) the NAb titer of HB02 vaccine against the HB02 strain on day 28/35/42 after immunization; (**D**) the NAb titer of Delta vaccine against the Delta strain on day 28/35/42 after immunization; (**E**) the NAb titer of Omicron vaccine against the Omicron strain on day 28/35/42 after immunization; error bars represent SEM. ** *p* < 0.01; *** *p* < 0.001 ((**B**–**D**), Mann–Whitney test).

Here, we prepared the different inactivated SARS-CoV-2 vaccines (HB02, Delta, and Omicron vaccine candidates) to study their immune effects. To confirm the effect of the vaccine candidates, the mice were immunized with the HB02, Delta, and Omicron vaccine candidates. Blood was collected to test the neutralizing antibody titers. The results show that the neutralizing antibody levels showed an upward trend in HB02, Delta, and Omicron inactivated-vaccine-immunized mice serum (Figure 1A). Additionally, the neutralization geometric mean titers (GMTs) of the serum from mice immunized with the HB02 vaccine for 42 days against HB02 were about 1156 (Figure 1B) and those for the Delta vaccine were 1079 (Figure 1C). Additionally, the GMTs of the Omicron vaccine against Omicron were about 1276 (Figure 1D). These results indicate that the vaccine candidates could lead to effective neutralizing antibodies.

**Figure 2:**
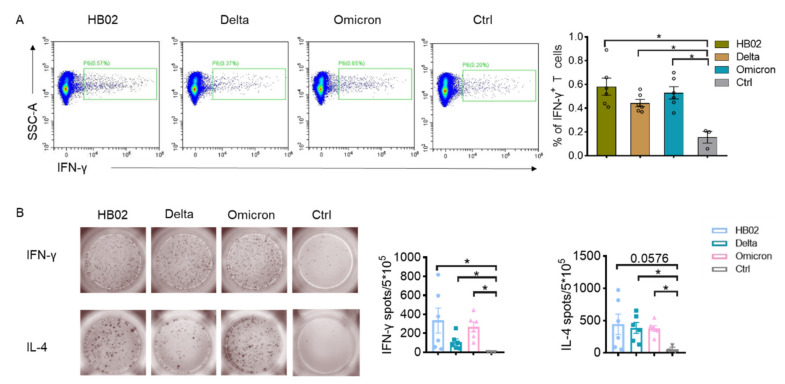
The vaccine-induced immune response. (**A**) Representative flow cytometry figures and percentages of IFN-γ-expressing T cells (gated in live CD45+ CD90+); (**B**) ELISPOT analysis of IFN-γ and IL-4 in spleens from vaccinated mice. Error bars represent SEM. * *p* < 0.05 ((**A**,**B**), Mann–Whitney test).

Here, we also tested the cellular immune response of the different vaccine candidates. The immune cells from the vaccinated mice spleen were analyzed. The results showed that different vaccine candidates could induce IFN-γ production by T cells, and there were no significant differences between these groups (Figure 2). This indicated that the inactivated vaccine could induce cellular immune response, which was not affected by the variants.

### 2.2. Metabolic Profiles

**Figure 3:**
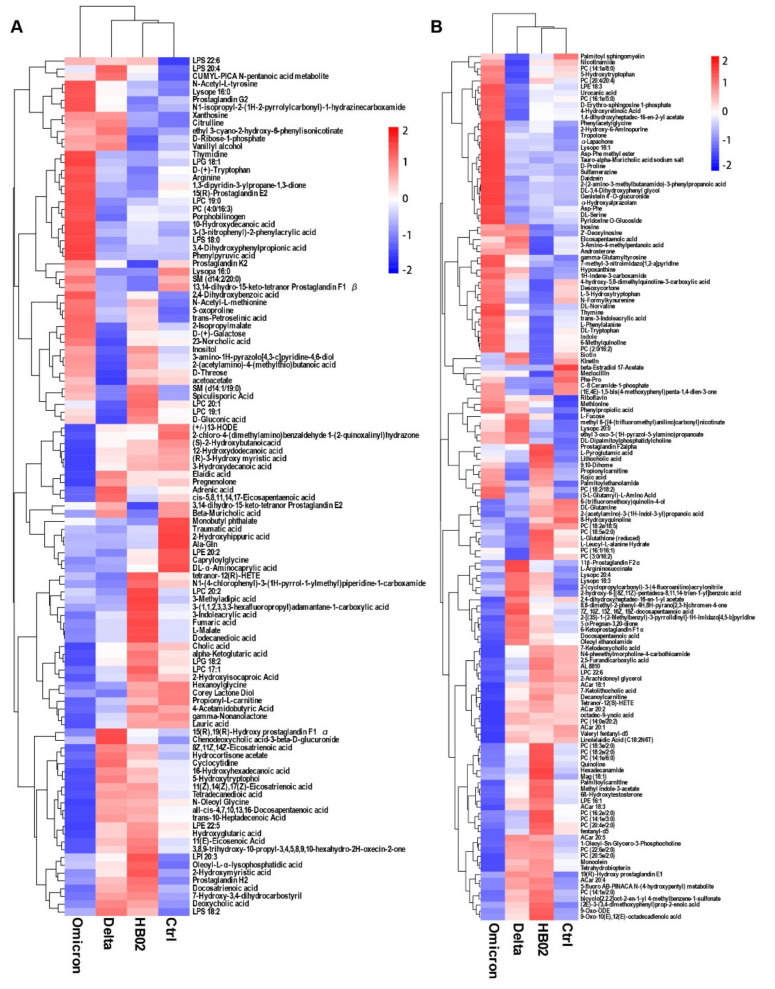
The change in the metabolism induced by vaccines. (**A**) The positive ion of the metabolism in the serum of the mice immunized with different vaccines on day 42. (**B**) The negative ion of the metabolism in the serum of the mice immunized with different vaccines on day 42. Numbers −2 to 2, z-values, represent the relative expression of differentially expressed genes.

In order to explain whether changes in amino acids could affect metabolomics and proteomics, we analyzed the metabolomics and proteomics of the serum from the mice immunized with the HB02, Delta, and Omicron vaccine candidates. Here, we detected 607 metabolites that were involved in cellular processes, environmental information processing, and organismal systems. The results show that the metabolic and protein profiles in the serum of mice immunized with different vaccines were very different (Figure 3A,B). The principal component analysis (PCA) also showed that the metabolic profiles were different in the HB02 and Omicron groups, mainly in terms of TCA cycle, pyruvate metabolism, and amino acid metabolism (Figure S1). Certain metabolites in TCA, such as acetyl-CoA, succinate, NAD, and α-ketoglutarate, could act as cofactors for epigenetic enzymes that enhance immune memory [12]. Moreover, the other metabolites were also changed. Deoxycholic acid (DCA) was upregulated after HB02 and Delta vaccine immunization, and it was secondary (or degenerated) bile acids [13]. It is a signaling molecule that acts on G protein-coupled bile acid receptor 1 (GPBAR1 or Takeda G-protein receptor 5) and the Farnesoid-X-Receptor (FXR). Both receptors are expressed by intestinal and liver macrophages, dendritic cells, and natural killer T cells. The activation of GPBAR1 and FXR can induce an anti-inflammatory response. The cytokine storm caused by SARS-CoV-2 infection is an important factor in organ damage and severe disease or death [14]. Therefore, the DCA induced by the vaccine could perhaps act as an immunomodulator. In addition to DCA, prostaglandin H2 (PGH2) is also upregulated. It is generated from prostaglandins, which are key lipid mediators that regulate physiological functions and inflammatory responses in health and disease [15]. Additionally, it is processed into the other prostaglandins [15,16]. Prostaglandins are a key mediator of immunopathology in chronic infections. However, these metabolites are present at lower levels in Omicron-vaccine-immunized mice. Additionally, phenylpyruvic acid and PGE2 are upregulated in Omicron-vaccine-immunized mice. These are essential homeostatic factors that can support the activation of dendritic cells and Th2 cells [17], which promote humoral immunity. Additionally, they can modulate chemokine production, inhibiting the attraction of proinflammatory cells [18,19,20], thus helping to avoid adverse reactions. Additionally, the Omicron-inactivated vaccines upregulate arginine in mice serum, and it has been reported that arginine can regulate the metabolic changes in activated T cells switching from glycolysis to oxidative phosphorylation and promote the generation of central memory-like cells. Microbiome-derived inosine is also upregulated by Omicron, and it has been reported that it is an alternative carbon source for CD8+ T cells and can modulate T-cell response [21,22]. Additionally, L-phenylalanine is also induced by Omicron vaccines, not HB02 and Delta vaccines. L-phenylalanine is associated with immune activation [23]. The antibody production induced by vaccines requires large amounts of amino acids. Through nontarget metabolite detection, many amino acids, such as arginine and proline, are upregulated; these are also different in the HB02, Delta, and Omicron groups. These results show that different SARS-CoV-2 variants induce different changes in metabolites, and HB02 and Delta have similar metabolic profiles, different from that of Omicron.

### 2.3. Proteomic Profiles

**Figure 4:**
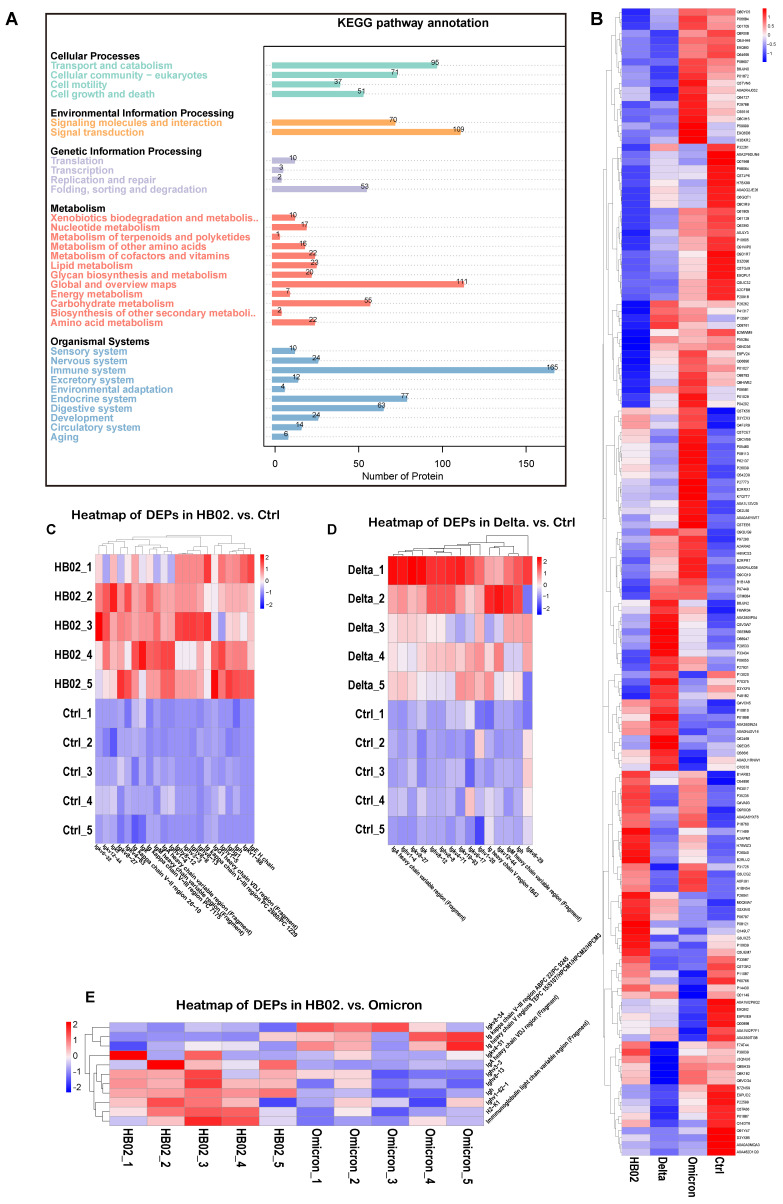
The change in the proteins induced by vaccines. (**A**) Biochemical metabolic pathway and signal transduction pathway analysis of proteins obtained by proteome sequencing using KEGG. (**B**) Heatmap analysis of DEPs from different groups. (**C**) Heatmap analysis of selected DEPs from HB02 vs. ctrl. (**D**) Heatmap analysis of selected DEPs from Delta vs ctrl. (**E**) Heatmap analysis of selected DEPs from HB02 vs. Omicron.

Besides metabolites, proteins were also confirmed. We identified 165 proteins related to the immune response (Figure 4A), including complementary systems and other immune-associated pathways. The results show that B1ARB3 (platelet endothelial cell adhesion molecule, PECAM-1) was upregulated after immunization with the HB02 vaccine (Figure 4B). Additionally, PECAM-1 is expressed in leukocytes, including T cells, B cells, and dendritic cells, and could be derived primarily from the protein’s integration of adhesion and signaling functions in the immune and vascular systems [24,25]. Q9EQI5 (chemokine subfamily B Cys-X-Cys) was induced by the Delta vaccine and was secreted by Th1 cells, mainly mediating cytotoxicity and local inflammation-related immune response to help in antibody production [26]. Additionally, comparing the control group, the HB02, Delta, and Omicron vaccines could induce immunoglobulin secretion (Figure 4C–E), supporting antibody production. However, the immunoglobulin secretion was also different in the HB02 and Omicron groups (Figure 4E), indicating that the different vaccines could induce different immune responses. Those results show that the HB02 and Omicron vaccines induced different metabolic and proteomic profiles, although the HB02 and Omicron strains had some amino acid site mutations. Amino acid changes affected the proteomics and metabolomics.

## 3. Discussion

Since the emergence of SARS-CoV-2 in 2019, SARS-CoV-2 has continued to mutate, and this is likely to continue, leading to the emergence of new mutants, especially those with altered spike proteins. The evolutionary trajectory of SARS-CoV-2 is still uncertain, and the genetic and antigenic characteristics of future variants cannot be predicted. Here, we used the HB02, Delta, and Omicron inactivated vaccine candidates to explore their protective effect and found that the respective vaccine candidates could lead to effective neutralizing antibodies. We also tested the cellular immune response. Besides IL-4, the different inactivated vaccines could induce IFN-γ secretion by T cells, which was tested by Flow and ELISPOT, and found no significant difference among these groups, a result consistent with previous research findings [27].

Proteomics and metabolomics were beneficial for the early events of the immune response [28,29] and adverse reaction detection [30], as well as providing a deeper understanding of immune cell responses. Proteomics and metabolomics provide valuable information about pathogen–host cell interactions. These two methods have accelerated the identification and detailed identification of new antigens that are potential candidates for vaccine developments [31]. In our study, we tested the metabolomics and proteomics in mice serum after immunization with different vaccines, which were HB02, Delta, and Omicron. We found that a large number of metabolites changed in the HB02-, Delta-, and Omicron-vaccine-immunized mice, but the Omicron-immunized group had a special metabolome compared with the other two groups, which may be because it is the most variable variant and did not develop from one of the earlier known SARS-CoV-2 variants [32]. Whether arginase, PGE2, and L-phenylalanine could be used as plasma metabolomic markers after omicron vaccine immunization deserves further study. The proteomic results showed that the expression of a large number of proteins related to antibody production in the serum of the three groups of immunized mice was significantly upregulated, indicating that the HB02, Delta, and Omicron vaccines could significantly induce humoral immune responses in mice, which was important for immune defense. Like the results of metabolomics, we also found that the serum proteomic changes in mice immunized with the Omicron vaccine were quite different from those of the other two groups, which further confirmed the specificity of the origin of the Omicron variant [33]. The omics analysis results of this study identified specific proteins and metabolites related to vaccine immunization, which provided a reference for screening candidate vaccine markers after immunization.

Furthermore, this paper correlates immune response and omics analyses to reveal how different variants differ across multiple dimensions. At the same time, we constructed the metabolic and protein profiles induced by different variants, providing a novel perspective on the omics differences induced by Omicron strains as well as guidance for screening candidate immune markers such as key proteins and metabolites. In addition, our findings provide a new direction for evaluating the immune response of vaccines or viruses.

## 4. Materials and Methods

### 4.1. Vaccine

The HB02, Delta, and Omicron vaccine candidates were prepared in the same way as previously described by Beijing Institute of Biological Products Company Limited [34]. Briefly, after virus culture harvest, β-propionolactone was added to the viral solution (ratio of 1:4000) at 4–8 °C, which could inactivate the virus. Then, after 20–24 h, it purified the inactivated virus. Finally, the vaccine was prepared by mixing the aluminum adjuvant (at a specified ratio) with the final purified virus.

### 4.2. Animal Models

All the mice (BALB/c, 17–19 g) were purchased from Beijing Vital River Laboratory Animal Technology Co., Ltd., Beijing, China, and maintained in a specific pathogen-free (SPF) environment at the Laboratory Animal Center of Beijing Institute of Biological Products Co., Ltd., Beijing, China. The total number of animals in each group is presented in Table 1.

### 4.3. Neutralization Assay

Mice were randomly divided into different groups and immunized with the vaccine candidates (0.5 mL/mice, intramuscularly) on Day 0 and Day 21. The blood was collected on Day 28, Day 35, and Day 42. The neutralization assay was based on the microplate CPE (micro-cytopathogenic efficiency) method described by Wang [34]. Briefly, the serum was inactivated in 56 °C water for 30 min. Then, the serum was diluted 4-fold, serially diluted 2-fold on a 1:4 basis to the desired dilution, and an equal volume of challenge virus solution with 100 CCID50 was added. The 0.1 mL/well cell suspension was cultured in a 37 °C CO_2_ incubator, and the results were collected on the 4th day.

### 4.4. Flow Cytometry

The spleens were collected on Day 42 after initial immunity and minced. Next, the spleen cells were lysed using RBC (Biolegend, San Diego, CA, USA, 420301). Immune cells were seeded at 1 × 10^6^ per well in 96-well plates and incubated with inactivated virus stock solution (concentration were 8 μg/well) in DMEM medium (Gibco, Thermo Fisher Scientific, Waltham, MA, USA, C11995500BT), which contained 1% penicillin-streptomycin (Procell, PB180120) and 10% FBS (Gibco, Thermo Fisher Scientific, Waltham, MA, USA), followed by BFA (Biolegend, San Diego, CA, USA, 420601) for 4 h. After the stimulation was complete, cells were harvested, blocked with CD16/32 antibody for 30 min at 4–8 °C, and then stained with an antibody against surface antigen using the appropriate antibody in blocking buffer on ice for 30 min. After surface staining, the cells were fixed with IC Fixation Buffer (eBioscience, San Jose, CA, USA). Fixable Viability Dye eFluor 506 was added to exclude the dead cells. Flow cytometry was performed on a CytoFLEX S instrument (Beckman, Brea, CA, USA). All cells were gated in live cells and analyzed to determine their levels of T cells (CD45+ CD90+) using the CytoFLEX S FlowJo software [27].

### 4.5. ELISPOT Assay

The IFN-γ and IL-4 produced by immune cells were tested with the Mouse IFN-γ precoated ELISPOT kit and the Mouse IL-4 precoated ELISPOT kit (Dakewe Group, Shenzhen, China). The detailed protocol was described by [27].

### 4.6. Metabolic Analysis

Metabolite extraction: samples were derived from mouse serum 42 days after immunization. We took 100 μL of sample into an EP tube and added 400 μL of 80% methanol. Then, the samples were incubated on ice for 5 min and centrifuged at 15,000× *g*, 4 °C, for 20 min. We diluted the supernatant to a final concentration of 53% methanol with LC-MS-grade water and transferred it to an Eppendorf tube, where it was centrifuged at 15,000× *g*, 4 °C, for 20 min.

LC-MS/MS system analysis: The supernatant was injected into the LC-MS/MS system. UHPLC-MS/MS analyses were performed using a Vanquish UHPLC system (Thermo Fisher, Germany) coupled with an Orbitrap Q ExactiveTM HF mass spectrometer (Thermo Fisher, Germany) in Novogene Co., Ltd. (Beijing, China). We injected the sample into a Hypesil Goldcolumn (100 × 2.1 mm, 1.9 μm) using a 17 min linear gradient at a flow rate of 0.2 mL/min. The eluents in the positive polarity mode were eluent A (which was 0.1% FA in Water) and eluent B (which was methanol). For the negative-polarity mode, eluent A was 5 mM ammonium acetate, pH 9.0; and eluent B was methanol. The solvent gradient settings were as follows: 2% B, 1.5 min; 2–100% B, 3 min; 100% B, 10 min; 100–2% B, 10.1 min; 2% B, 12 min. The Q ExactiveTM HF mass spectrometer was operated in positive/negative mode, spray voltage 3.5 kV, capillary temperature 320 °C, sheath gas flow 35 psi, assist gas flow 10 L/min, S-lens RF level 60, and assist gas heater temperature 350 °C.

Data analysis: The heatmaps were plotted with the R package Pheatmap and normalized to the metabolite data using z-score.

### 4.7. Proteomic Analysis

Sample processing: First, we removed high-abundance proteins. Brifely, samples were derived from mouse serum 42 days after immunization. We added BioRAD proteominer beads and blood (serum, plasma) sample to a 1.5 mL centrifuge tube and mixed on a vertical rotating mixer for 2 h. The mixture was centrifuged at 10,000× *g* for 5 min at 4 °C and the beads were washed repeatedly with wash buffer. We then added 0.4 mL 1% TFA to the washed beads, followed by 10 min of mixing repeatedly. After this, we took out the supernatant and repeated twice for elution. The supernatant was frozen into a dry powder in a freeze concentrator. The pellet was dissolved using dissolution buffer (8 M Urea, 100 mM TEAB, pH 8.5). Then, the solution was reduced with 10 mM DTT for 1 h at 56 °C, and subsequently alkylated with sufficient iodoacetamide for 1 h at room temperature in the dark. After that, the sample was tested to determine the protein quality. Next, we took the protein sample; added DB protein lysate, trypsin, and 100 mM TEAB buffer; mixed well; digested at 37 °C for 4 h; and then added trypsin and CaCl2 to digest overnight. After adjusting the pH and centrifuging for 5 min, we took the supernatant and slowly passed it through a C18 desalting column, then used 0.1% formic acid and 3% acetonitrile to wash it three times continuously. Then, we added an appropriate amount of eluent, collected the filtrate, and freeze-dried it.

Data-independent acquisition mode (DIA) detection: The precolumn of the EASY-nLCTM 1200 nanoliter UHPLC system was a self-made precolumn (4.5 cm × 75 μm, 3 μm), and the analytical column was a self-made analytical column (15 cm × 150 μm, 1.9 μm). The mass spectrometer used was Q ExactiveTM HF-X, the ion source used was Nanospray Flex™ (ESI), the ion spray voltage was set to 2.1 kV, the ion transfer tube temperature was 320 °C, and the mass spectrometer was in DIA and could generate mass detection data.

Data analysis: We used the software Proteome Discoverer 2.2 (PD2.2, Thermo) to search for data in DDA scan mode. The statistical analysis of the protein quantification results was performed using the *t*-test, and the proteins with a significant difference in quantification between the experimental group and the control group (*p* < 0.05, |log2FC| > * (FC > * or FC < * [fold change, FC]) were defined as differentially expressed protein (DEP).

### 4.8. Statistics

The statistical analyses were performed using GraphPad Prism 8. In comparisons between the two groups, a two-tailed Mann–Whitney test was used to determine significance. Error bars represent SEM. * *p* < 0.05; ** *p* < 0.01; *** *p* < 0.001.

## 5. Conclusions

Our study found that the HB02 and Omicron inactivated vaccine candidates could protect the body against the respective strains of SARS-CoV-2 and induce a cellular immune response. Furthermore, we found the specific metabolic and proteomic differences in the different vaccine candidates using the approach of omics analysis in systems vaccinology. Most of the altered metabolites and proteins have been previously reported as biomarkers of immune response. These insights provide new directions for the immune evaluation of vaccines and could serve as a guide to novel strategies for vaccine design.

Our study used mice as model. In future work, to further analyze the relationship between immune response and metabolomics or proteomics, suitable human volunteers should be selected to further clarify the mechanism.

## Figures and Tables

**Table 1:** Total number of animals in each group.

Experiment	Group	Total Number	Male	Female
Neutralization analysis	HB02	10	5	5
	Delta	10	5	5
	Omicron	10	5	5
Immunoassay (FACS, ELISPOT)	HB02	6	3	3
	Delta	6	3	3
	Omicron	6	3	3
Omic analysis	HB02	5	3	2
	Delta	5	3	2
	Omicron	5	3	2

## Data Availability

Not applicable.

## Supplementary Materials

The following supporting information can be downloaded at: https://www.mdpi.com/article/10.3390/ijms231810644/s1.
