# Supplementary material for: Metabolic and Proteomic Profiles Associated with Immune Responses Induced by Different Inactivated SARS-CoV-2 Vaccine Candidates
Source: Int J Mol Sci. 2022 Sep 13;23(18):10644. doi: 10.3390/ijms231810644 (PMC9503298; doi:10.3390/ijms231810644)
Supplement: Supplementary file 1 [file ijms-23-10644-s001.zip › ijms-1874292-supplementary.pdf]

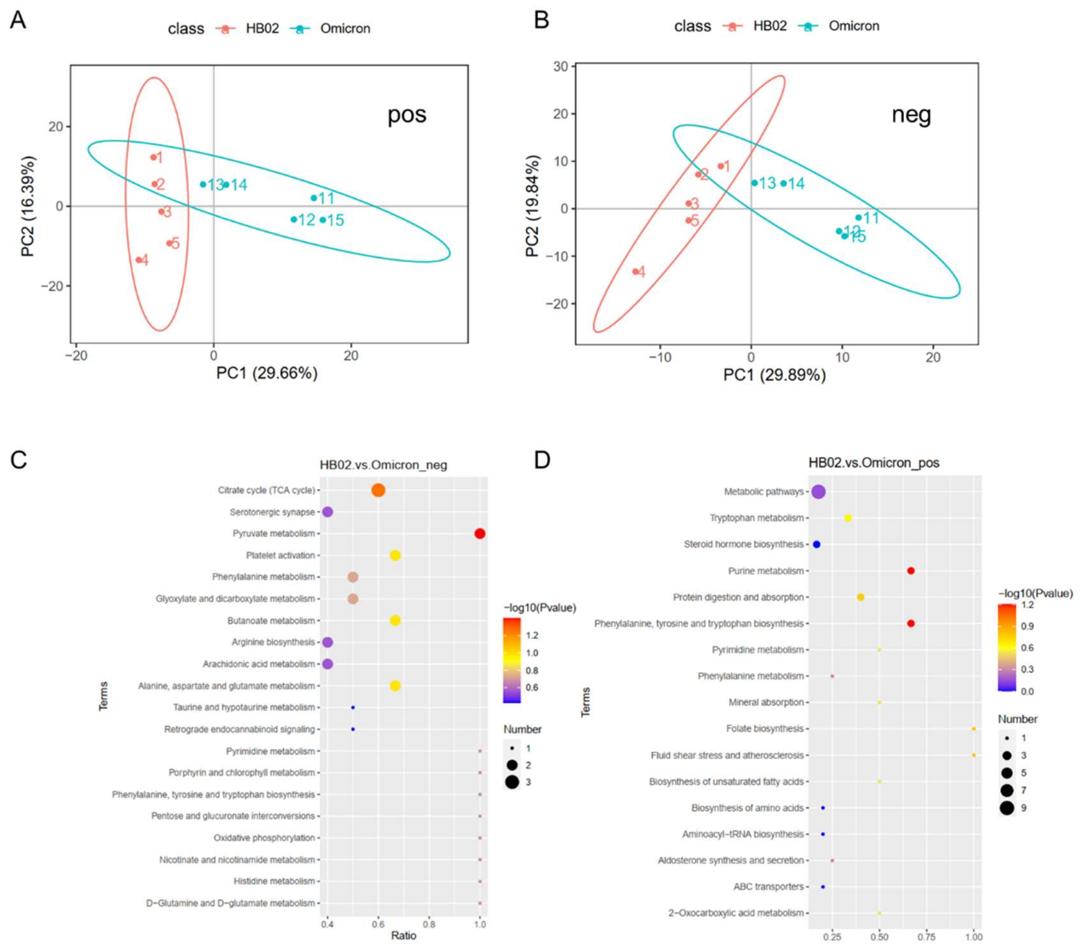


**Figure S1. Principal Component Analysis and Met KEGG Enrichment in HB02 and Omicron group.**

(**A**,**B**) PCA of metabolites in HB02 and Omicron group;

(**C**,**D**) KEGG analysis of HB02 and Omicron group.
